# The skin I live in: Pathogenesis of white-nose syndrome of bats

**DOI:** 10.1371/journal.ppat.1012342

**Published:** 2024-08-29

**Authors:** Marcos Isidoro-Ayza, Jeffrey M. Lorch, Bruce S. Klein

**Affiliations:** 1 Department of Pediatrics, Medicine and Medical Microbiology and Immunology, School of Medicine and Public Health, University of Wisconsin-Madison, Madison, Wisconsin, United States of America; 2 U.S. Geological Survey, National Wildlife Health Center, Madison, Wisconsin, United States of America; University of Georgia, UNITED STATES OF AMERICA

## Abstract

The emergence of white-nose syndrome (WNS) in North America has resulted in mass mortalities of hibernating bats and total extirpation of local populations. The need to mitigate this disease has stirred a significant body of research to understand its pathogenesis. *Pseudogymnoascus destructans*, the causative agent of WNS, is a psychrophilic (cold-loving) fungus that resides within the class Leotiomycetes, which contains mainly plant pathogens and is unrelated to other consequential pathogens of animals. In this review, we revisit the unique biology of hibernating bats and *P*. *destructans* and provide an updated analysis of the stages and mechanisms of WNS progression. The extreme life history of hibernating bats, the psychrophilic nature of *P*. *destructans*, and its evolutionary distance from other well-characterized animal-infecting fungi translate into unique host–pathogen interactions, many of them yet to be discovered.

## Introduction

Bats are vital for our planet’s biodiversity and ecosystems. They are the second-most species-diverse group of mammals after rodents [1]. Forty-seven species of bats occur in the United States of America (USA) and Canada, and due to high population sizes and the tendency for many species to congregate in large numbers, they are among the most locally abundant mammals in North America [2]. They are vital in maintaining balanced habitats and agricultural systems, providing nutrients through their guano, and consuming vast amounts of insects, including pests that damage wild plants and crops [3,4]. Bats also affect human health by predating disease-vectoring insects, including mosquitoes of the genus *Culex*, which carry viral pathogens such as West Nile or St. Louis encephalitis viruses [5,6]. Despite their contributions to biodiversity, ecosystem function, human health, and the economy, North American bat populations have been experiencing large declines, with 31 percent of the species at risk or potentially at risk of extinction [2].

**Fig 1:**
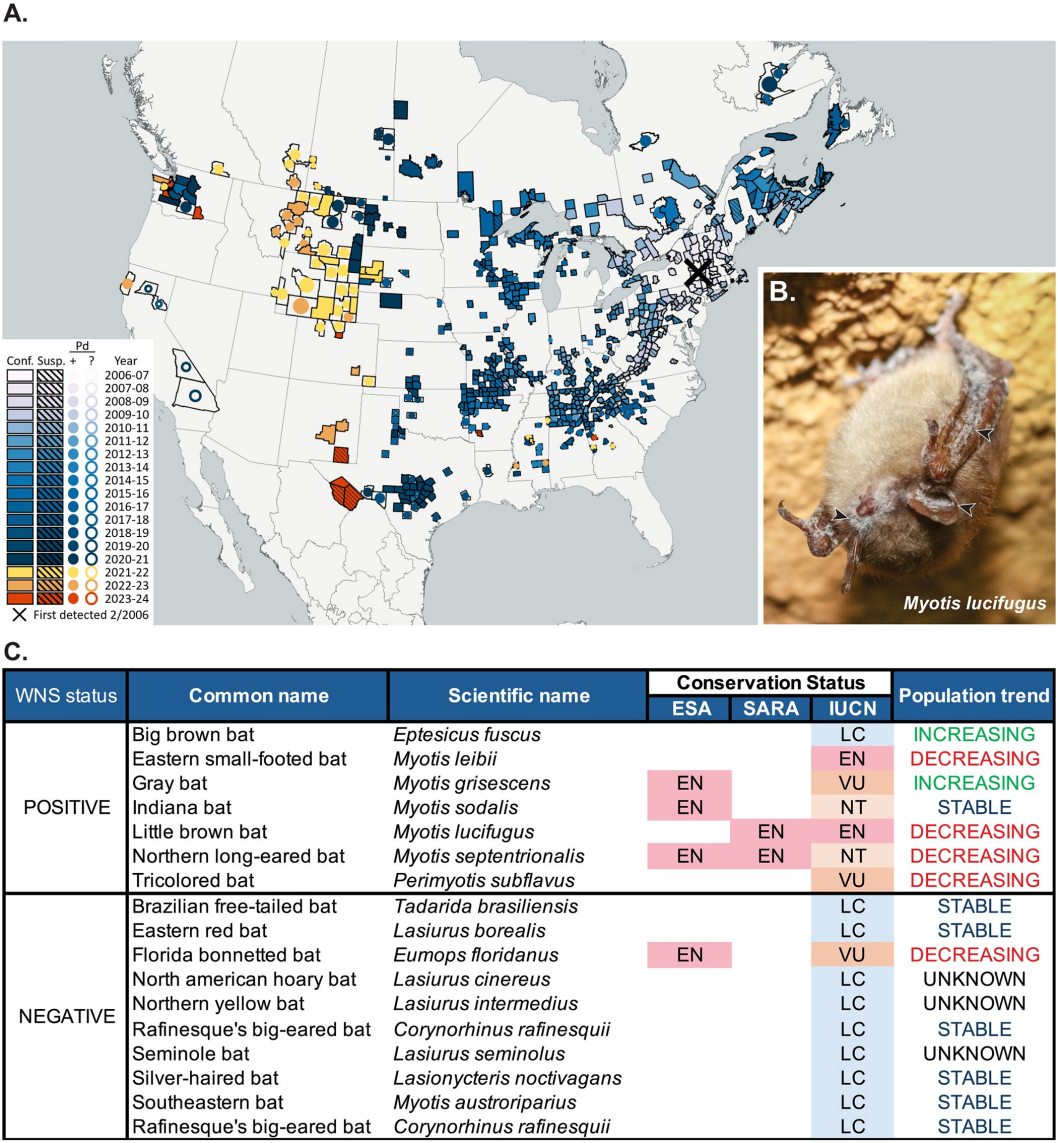
Distribution and impact of WNS on North American bats. **(A)** Map of distribution of WNS in North America (2006–2024). Conf., WNS confirmed, Susp., WNS suspect; Pd+, *Pseudogymnoascus destructans* detected; Pd?, *P*. *destructans* suspect [126]. **(B)** Little brown bat (*Myotis lucifugus*) with characteristic macroscopic WNS lesions consisting of white fuzzy-flaky material (i.e., *P*. *destructans* hyphae) on the skin of the wing, pinnae, and around the nose. Photo by Heather Kaarakka, Wisconsin DNR. **(C)** WNS status based on histopathology and quantitative PCR detection of *P*. *destructans* [79,127], conservation status (ESA, SARA, IUCN), and population trends (IUCN) of continental bat species of the eastern USA and Canada. ESA, US Endangered Species Act [128]; SARA, Canada Species at Risk Act [129]; IUCN, The International Union for Conservation of Nature Red List of Threatened Species [130]; EN, endangered; VU, vulnerable; NT, near threatened; LC, least concern.

White-nose syndrome (WNS) is one of the most pressing threats for hibernating species of bats in the USA and Canada and one of the most devastating infectious diseases of wild mammals of the last century [2,6,7]. The causative agent, *Pseudogymnoascus destructans*, is a psychrophilic (cold-loving) fungus that invades and parasitizes bat skin [8–10]. Since its first report in North America in February 2006 (Albany County, New York), WNS has killed millions of bats on the continent, with over 95% declines in some species and extirpation of entire populations [6,7,9,11]. The disease has been confirmed in 40 USA states and 9 Canadian provinces and is spreading westward across North America [6] (**Fig 1**). The need to mitigate the impacts of this disease has translated into calls for the development of evidence-based treatments and a better understanding of the mechanisms involved in disease progression or pathogenesis of WNS. In this article, we provide an updated outlook on WNS pathogenesis by describing and discussing the particularities of host and pathogen, the forms of transmission, and the different phases of host–pathogen interactions, and by identifying factors influencing disease severity and proposing future research directions.

## The host

### Bat hibernation

WNS exclusively affects hibernating bats [6]. Insectivorous bats in North America evolved to undergo hibernation to cope with food scarcity and harsh winter conditions [12]. During hibernation, bats enter prolonged periods of torpor of approximately 2 to 3 weeks, during which their body temperature approximates that of the roosting site (approximately 1 to 16°C) [12,13]. During torpor, bats, like other hibernating mammals, are metabolically dormant, with virtual cessation of transcription and translation [14,15]. Hibernating bats are also immunologically depressed, with severe leukopenia involving neutrophils, monocytes, and lymphocytes, due to transient sequestration of these immune cells in lungs, liver, or lymphoid organs and limited neutrophilic activation [16–20]. Following each period of torpor are shorter arousals of approximately 1 to 3 h characterized by euthermia with body temperature approaching 37°C and rapid metabolic and immunological reactivation [12–19,21]. In this context of immune suppression, torpid bats rely on their long periods of low body temperature and metabolic dormancy to keep microbial pathogens in check [22]. Most bacterial pathogens of mammals are mesophilic and, therefore, unable to replicate at temperatures below 5°C, while viruses depend on the host’s metabolic machinery for replication [22–25]. Paradoxically, the long torpor bouts and short arousals characteristic of bat hibernation render hibernating bats more susceptible to a uniquely adapted, cold-loving pathogen such as *P*. *destructans*.

### Bat skin

Most pathological changes related to WNS involve the skin of the wing (plagiopatagia) and tail (uropatagia) membranes [26]. Patagial skin is thin, virtually devoid of hair, and consists of 2 epidermal layers separated by a thin dermal layer. The non-keratinized part of the epidermis is made of 1 to 2 layers of keratinocytes, while the stratum corneum has up to 10 layers of thin corneocytes (terminally differentiated keratinocytes) [27,28]. Other cells present in the epidermis are melanocytes, antigen-presenting Langerhans cells, and T lymphocytes [29]. The dermis is characterized by a scaffold of fibroblasts, collagen, and elastic fibers supporting lymphatic and blood vessels, neurons, and specialized immune cells such as dendritic cells, macrophages, lymphocytes, natural killer, and mast cells [27–29]. The thin nature of the patagial skin results from functional adaptations to flight, thermoregulation (heat dissipation), and gas exchange (O_2_ and CO_2_) [26,30]. Moreover, bats are highly susceptible to dehydration during hibernation and losses through the wing skin account for 99% of the total water loss [26]. Therefore, epidermal disruption of the patagia during WNS ultimately translates into disruption of the host’s physiology.

## The pathogen

**Fig 2:**
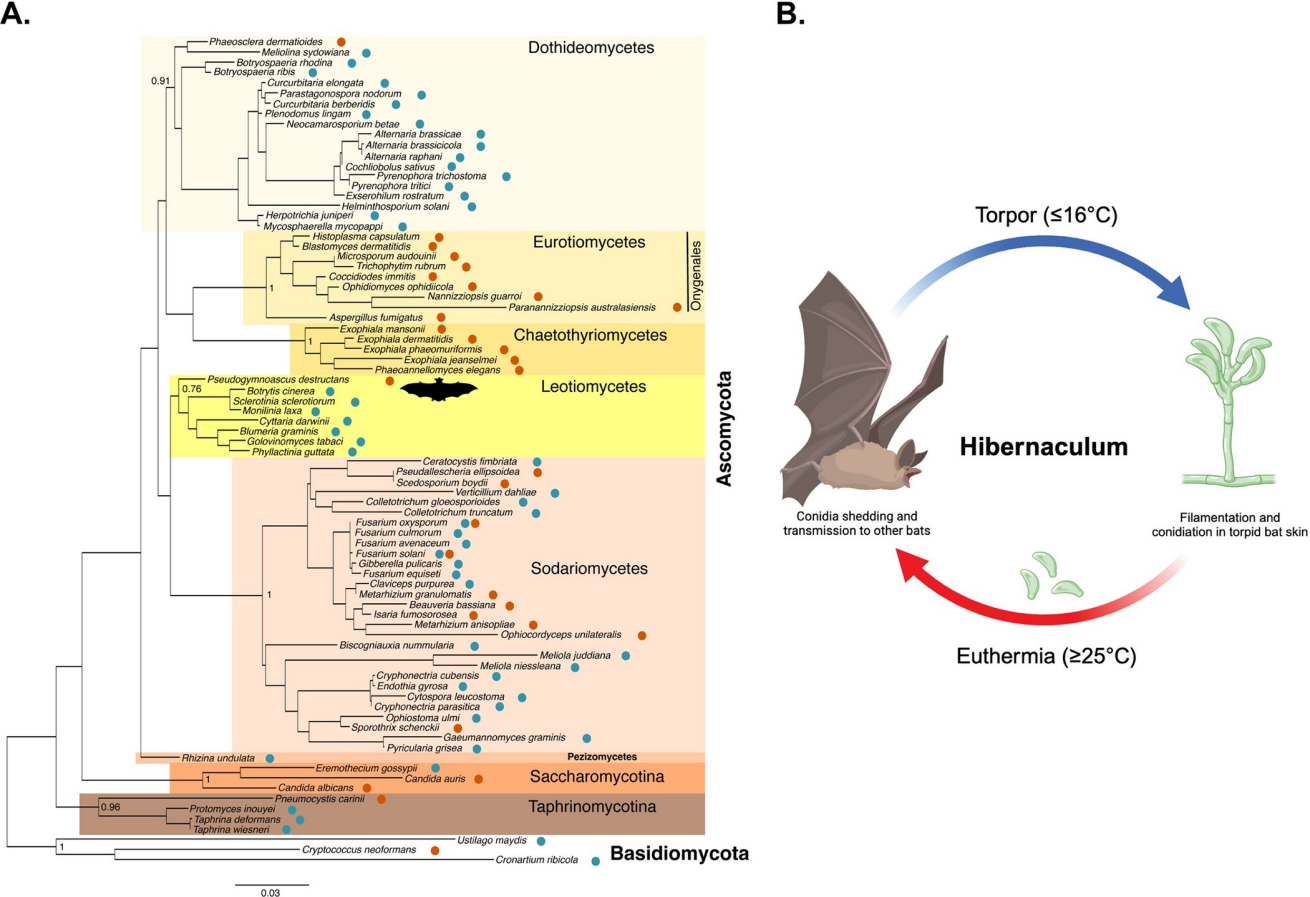
Phylogeny and life cycle of *Pseudogymnoascus destructans* in hibernacula. **(A)** Phylogenetic tree showing the major taxonomic groups with representative taxa of pathogenic ascomycete fungi. Plant pathogens are denoted by blue dots, while animal (including insect) pathogens are marked with orange dots. Note that *P*. *destructans* (marked with a bat icon) resides within the class Leotiomycetes that otherwise contains mostly plant pathogens and is not closely related to other consequential pathogens of humans and animals (e.g., Onygenales, *Candida* spp.). Support values from a maximum likelihood analysis are presented for each major group and the tree is rooted with representative pathogenic fungi from the division Basidiomycota; taxa included in the tree are based on Berbee with some modifications [33]. **(B)** *P*. *destructans*’ life cycle in hibernacula. Transmission of conidia (dormant form of the fungus) from the hibernacula environment to bats occurs while bats are active (euthermia). Conidia germinate and colonize the skin of torpid bats. Subsequent hyphal conidiation leads to shedding of conidia into the environment. Conidia remain viable in the hibernacula environment until the following winter, which perpetuates the cycle of transmission. Figure designed using BioRender (Agreement # BH271RUAM8).

*P*. *destructans* is a filamentous fungus in the Division Ascomycota, Class Leotiomycetes, Order Thelebolales [9,31,32]. Other than *P*. *destructans*, Leotiomycetes includes mostly plant pathogens with no relevant animal pathogens, making this fungus unique among other animal-infecting fungi (**Fig 2A**) [32,33]. Three distinct *P*. *destructans* clades have been identified and geographically grouped in Far-East Asia, Central Asia, and Europe [34]. *P*. *destructans* is believed to have been established in North America through a single introduction event followed by clonal spread from Europe, where it coexisted with hibernating bats for millennia without causing known mortalities or population declines [34]. Supporting this hypothesis, experimental infection of North American bats (*Myotis lucifugus*) with a European strain of *P*. *destructans* caused WNS with mortality and severity comparable to those caused by a North American strain [35]. *P*. *destructans* is a haploid heterothallic fungus with 2 mating types (MAT1-1 and MAT1-2) needed for sexual reproduction. Although both genotypes have been identified in Europe, only MAT1-1 has been found in North American isolates [34]. Therefore, only the asexual lifecycle of *P*. *destructans* is known to occur in North America, with slender, thin-walled hyphae producing curved (banana-shaped) melanized conidia [9,10] (**Fig 2B**). Environmental factors are important modulators of *P*. *destructans*’ virulence, and WNS is more likely to affect bats that roost in warmer and more humid hibernacula and microenvironments [36,37]. *P*. *destructans* is a psychrophilic fungus that grows at temperatures ranging from 1 to 20°C (maximum growth rate between 12 and 16°C), which is within the temperature range of torpid bats and their hibernacula [38–40]. Above 16°C, *P*. *destructans* undergoes pronounced morphological changes suggestive of heat stress, including increased septation and thickening of hyphae, as well as conidiation with altered conidial shapes and production of arthrospores and chlamydospore-like structures [38]. *P*. *destructans*’ germination rate, mycelial growth, and conidiation also increase with high relative humidity (RH; 70.5% to 81.5% at 13°C), whereas RH <70% restricts filamentation (but not conidiation) [41].

## Transmission

In the absence of bats during late spring-early fall, *P*. *destructans* survives in the substrate of the hibernacula, persisting as a dormant conidium or potentially growing as a saprophyte (environmental decomposer) [42] (**Fig 2B**). This saprophytic capacity of *P*. *destructans* is limited, compared with closely related nonpathogenic *Pseudogymnoascus* spp., due to a marked reduction in carbohydrate-utilizing enzymes (CAZymes) and predicted secretome, likely as a consequence of a tradeoff from its acquired parasitic lifestyle [43–45]. During the autumn swarm, *P*. *destructans* is thought to be transmitted to non-contaminated bats through contact with cave substrates or by interaction with contaminated bats [8,46,47] (**Fig 2B**). During the autumn swarm period, highly interactive bats might carry viable *P*. *destructans* spores to different regional hibernacula, contributing to the geographic spread of the pathogen [47,48]. Further transmission can also occur during hibernation when infected bats transmit *P*. *destructans* to uninfected bats during interbout arousals [47] (**Fig 2B**). On the skin of torpid bats, *P*. *destructans* enters a phase of faster growth and a parasitic lifestyle that is lacking in its close saprophytic relatives, shedding vast amounts of conidia into the environment by the end of the hibernation [43,49,50] (**Fig 2B**). Those conidia remain viable in the cave or mine environment until subsequent winters, perpetuating the cycle of reinfection [51,52] (**Fig 2B**). Importantly, *P*. *destructans*’ environmental reservoir makes disease transmission possible even at low host densities, increasing the likelihood of population effects [46]. Surviving bats clear the infection upon emergence from hibernation, and *P*. *destructans* detection in bat skin during summer months is relatively low [53–55]. Still, residual conidia can survive for months on their fur at elevated temperatures (24 to 30°C), making transmission of *P*. *destructans* at maternity colonies or summer roosts unlikely but theoretically possible [56].

## The disease

### *P*. *destructans* interactions with skin

**Fig 3:**
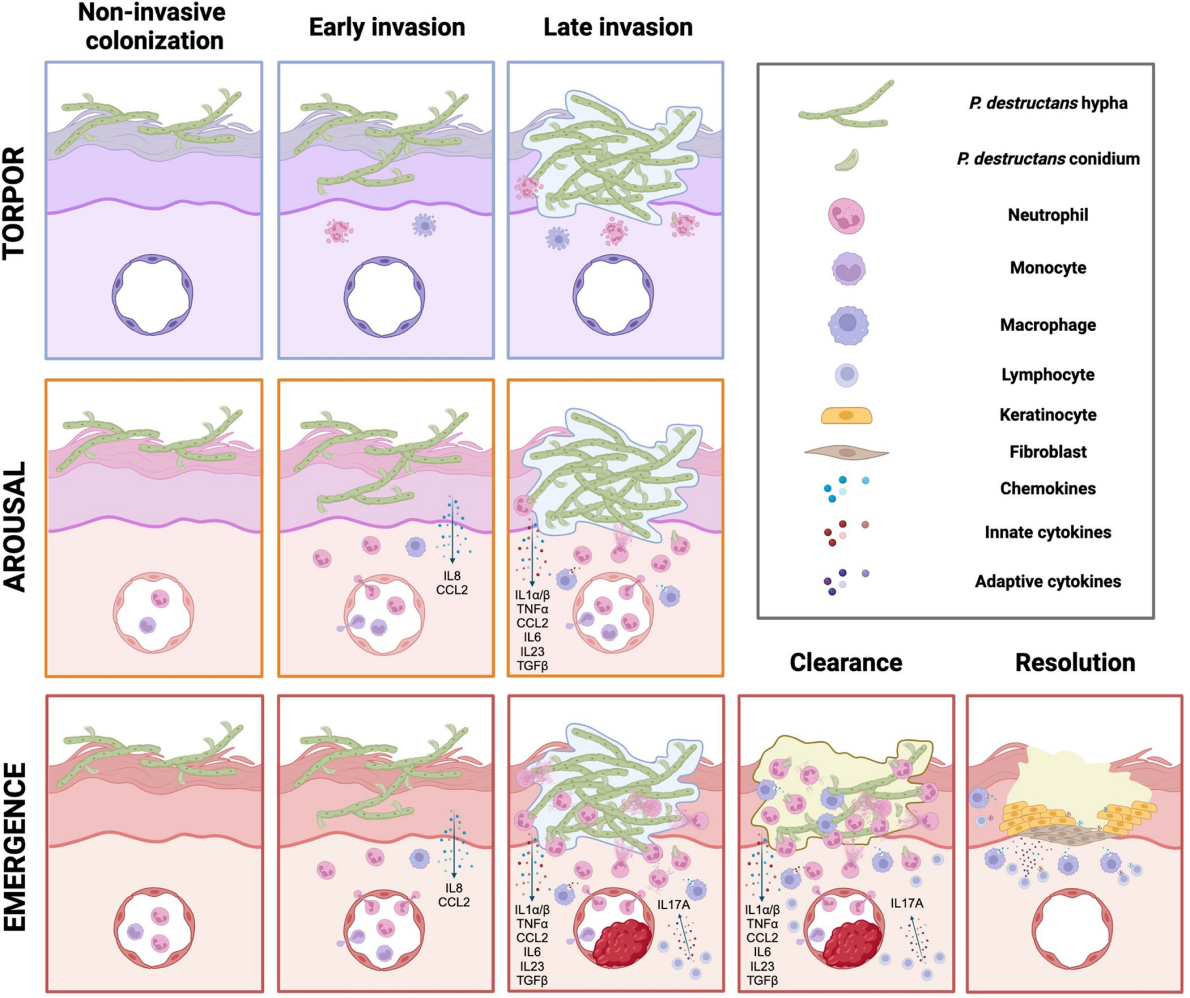
Proposed model of WNS pathogenesis. Our proposed model of WNS pathogenesis is divided into the following phases: (1) Noninvasive colonization involves superficial colonization of the epidermis (stratum corneum) without recruitment of inflammatory cells regardless of the hibernation phase (torpor, arousal, or emergence). (2) Early invasion consists of non-damaging entry into epidermal keratinocytes (deeper epidermis). Infected keratinocytes release chemokines during the euthermic periods (arousal or emergence) that might lead to recruitment of small numbers of phagocytes into the site of infection. Limited antimicrobial activity by local and newly recruited immune cells is expected at this stage given the intracellular location of *P*. *destructans* and the absence of cell damage signaling (e.g., alarmins). (3) Late invasion is characterized by increased *P*. *destructans* burden in the epidermis that leads to cell damage and replacement by biofilm-like matrix-embedded fungal clusters (cupping lesions) and up-regulation, during euthermia, of alarmins, chemokines and cytokines known to promote Th17 antifungal responses. The short duration of arousals likely precludes further recruitment of myeloid and memory T cells, which could partially explain the lack of IL17A up-regulation detected in these late invasion sites in aroused bats. Memory Th17 cells are likely recruited after a longer euthermic period (emergence) leading to a more robust antifungal inflammatory response that might lead to IRIS, thrombosis and ischemic necrosis of infected tissue. (4) Clearance and resolution happen if bats survive the hibernation period and the IRIS by eliminating the fungus and repairing the damaged tissue. Figure designed using BioRender (Agreement # XE271RUHMM).

The interactions between *P*. *destructans* and bat skin throughout the infection and disease process can be divided into the following phases: (1) noninvasive colonization; (2) early non-damaging invasion; (3) late damaging invasion; and (4) pathogen clearance and resolution (**Fig 3**).

**Fig 4:**
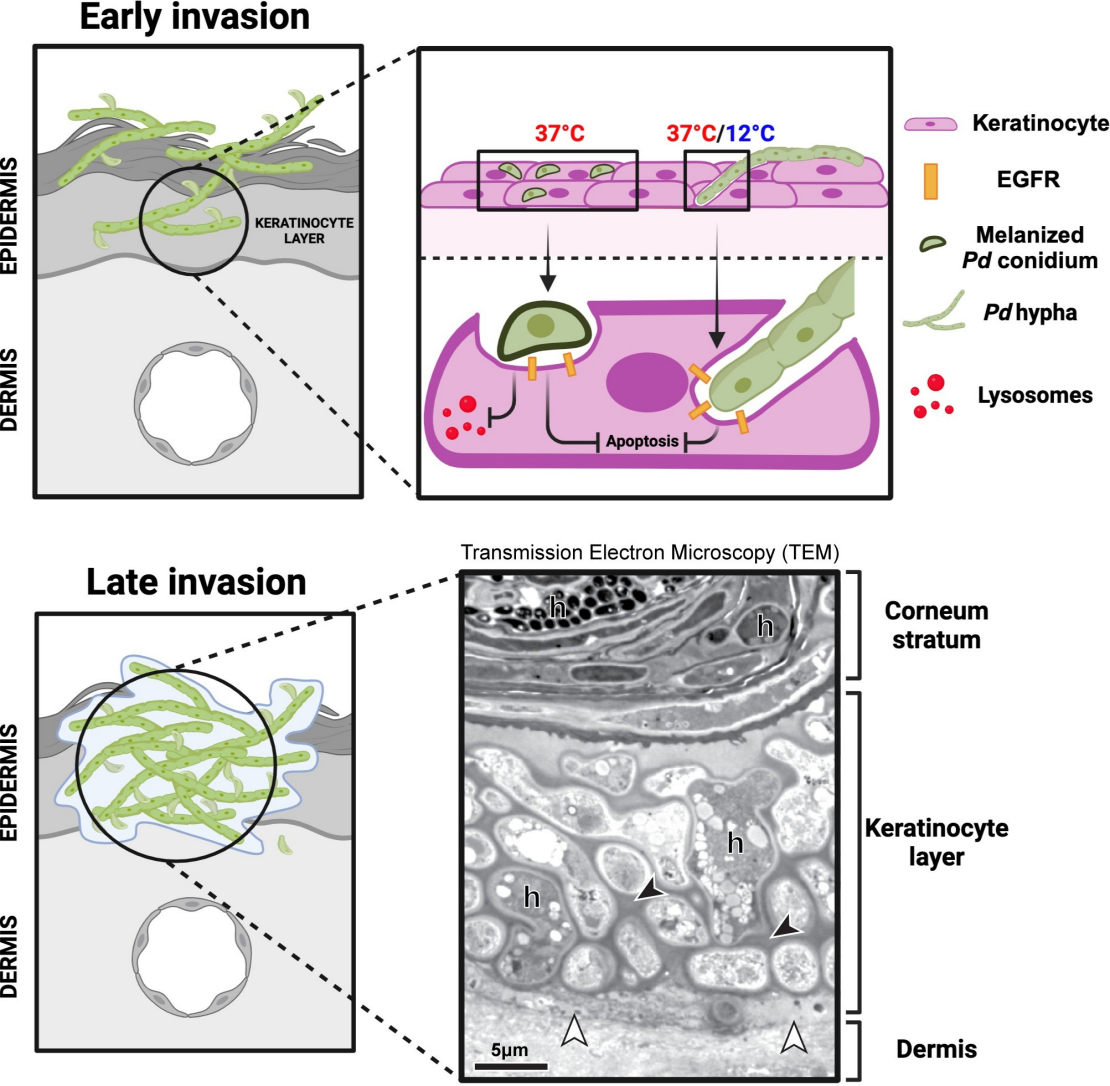
Pathogenic features of *P*. *destructans*’ skin invasion. During early invasion, *P*. *destructans* hyphae enter bat keratinocytes by EGFR-mediated endocytosis or active penetration at euthermic- (37°C) or torpor-like temperatures (12°C), respectively, while *P*. *destructans* conidia enter bat keratinocytes by EGFR-mediated endocytosis at euthermic-like temperatures. Invading *P*. *destructans* blocks apoptosis of infected keratinocytes and 1,8-dihydroxynaphthalene (DHN) melanin coating on conidia inhibits acidification and maturation of fungal containing endosomes favoring intracellular survival. During late invasion, tightly clustered pleomorphic hyphae (h) embedded in electrodense biofilm-like matrix (black arrowheads) replace dead keratinocytes (white arrowheads) forming cupping lesions. “Late invasion” transmission electron microscopy picture taken from the skin of a WNS–positive *Myotis lucifugus* previously sampled in Isidoro-Ayza and Klein [59]. Notice that superficial hyphae are narrower than those in deeper layers of the skin. Figure designed using BioRender (Agreement # MO271RUM95).

**1) Noninvasive colonization** starts with the adhesion of *P*. *destructans* conidia to the surface of the stratum corneum, probably mediated by hydrophobic interactions with glycosylphosphatidylinositol- (GPI-) anchored proteins to components of the extracellular matrix (ECM) [57,58]. Under the right conditions, during torpor, *P*. *destructans* conidia swell and germinate, forming a germ tube and hyphae that progress between corneocytes, likely feeding on the lipid-rich ECM of the stratum corneum while utilizing a repertoire of lipases and proteases [32,42,59–61]. Unlike dermatophytes, *P*. *destructans* is not known to be keratinolytic [42]. However, the secretion of aspartyl or serin proteases like Destructin-1 might contribute to the breakdown of ECM and corneodesmosomes (corneocyte’s intercellular junctions) to further colonize the stratum corneum [60]. Whether these enzymes are constitutively or differentially expressed depending on host factors or temperature shifts remains uncertain [50,62]. Germination, filamentation, and hyphal progression will be interrupted upon each arousal when skin temperature rises above 20°C. Colonization of the stratum corneum without further invasion into the non-cornified keratinocyte layer represents the dominant feature of *P*. *destructans*’ infection of less susceptible bat species (e.g., *Eptesicus fuscus* or *Myotis myotis*) [63–65].
**2) Early non-damaging invasion** of the epidermis transpires once *P*. *destructans* has breached the corneum stratum and initiates the invasion of non-cornified keratinocytes [59]. *P*. *destructans* can invade bat keratinocytes during both phases of hibernation (i.e., torpor and arousal) [59] (**Fig 4**). Invasion can ensue by fungal-mediated active hyphal penetration during the torpid phase when skin temperature is optimal for fungal growth (≤16°C) and host cells are metabolically dormant. Alternatively, *P*. *destructans* hyphae and conidia can passively enter bat keratinocytes through induced endocytosis (host-mediated) during arousals when *P*. *destructans* is inactive and host cells reactivate [59]. Both active hyphal penetration during torpor and induced endocytosis of hyphae and conidia during arousal are mediated by the transmembrane tyrosine kinase epidermal growth factor receptor (EGFR) [59]. EGFR facilitates the adhesion of *P*. *destructans* hyphae to bat keratinocytes at torpor-like temperatures (12°C) and endocytic uptake of *P*. *destructans* with pseudopodia formation by keratinocytes at euthermic-like temperatures (37°C) [59].

Keratinocytes are professional endocytic cells that sample the skin environment and kill internalized microbes by enzymatic digestion in acidic endolysosomes [66–68]. Remarkably, endocytosed *P*. *destructans* conidia survive inside the keratinocytes during arousal. In particular, the intracellular killing of *P*. *destructans* conidia is thwarted by its 1,8-dihydroxynaphthalene (DHN) melanin-containing surface coat, which inhibits endolysosome maturation and acidification [59]. Upon returning to torpor-like conditions, conidia germinate and colonize neighboring host cells [59]. Notably, the germination rate of intracellular conidia is significantly higher than that of extracellular conidia. This advantageous intracellular lifestyle indicates a potential biotrophic behavior of *P*. *destructans* during early skin invasion similar to some fungal plant pathogens [32,69] and might shield the fungus from antimicrobial factors secreted into the extracellular space by keratinocytes or other epidermal immune cells [59].
  During this early invasive phase, the invaded epithelium remains viable [59]. This lack of cell damage is partially explained by *P*. *destructans*’ nondisruptive penetration of the epithelial cells, which forms transcellular tunnels while preserving the epithelial plasma membranes, as previously described for *Candida albicans* and *Aspergillus fumigatus* [59,70,71]. In addition, invading *P*. *destructans* inhibits the infected keratinocytes’ programmed cell death (apoptosis) [59]. This anti-apoptotic effect requires viable *P*. *destructans* and direct interaction between *P*. *destructans* and epithelial cells, indicating that it is not mediated by secreted factors [59]. The epithelial invasion that characterizes this phase of WNS does not kill host cells and is reminiscent of the invasive commensalism described in early *A*. *fumigatus* or *C*. *albicans* invasion of epithelial cells before secretion of cytotoxic gliotoxin and candidalysin, respectively [70–72].
  The host immune response during this early invasive phase is consistent with the expected response of epithelial cells that recognize the presence of nonpathogenic fungal commensals. This response is restricted to arousal-like temperatures (37°C) in bats and consists of mild up-regulation of genes encoding chemokines such as CCL2 and IL8 without up-regulating cell damage-associated mediators (alarmins) such as IL1α, IL1β, or TNFα [59,73–75]. The gene encoding cycloxygenase-2 (COX2), a key enzyme for synthesizing proinflammatory eicosanoids like PGE_2_, is significantly up-regulated during early invasion of bat epithelium [59,76]. However, none of its proinflammatory products are detected, indicating a posttranscriptional blockade of this pathway likely due to lack of calcium flux-induced activity of phospholipase A2 [77]. These nonproductive, milder responses to the early infection likely represent the ability of epithelial cells to discriminate between commensal and pathogenic microbes [78]. This hyporesponsive mode might be essential for a tolerant energy-saving response by the host. However, the pathogen might also benefit from it to colonize bat skin further without facing host opposition.
**3) Late damaging invasion** occurs following increased fungal burden in the epidermis (**Fig 4**). *P*. *destructans* hyphae tightly cluster within the epidermis, forming cupping lesions embedded within a biofilm-like matrix and necrotic debris, likely accumulating secreted cytotoxic *P*. *destructans*’ metabolites (e.g., riboflavin) and hydrolytic enzymes (e.g., Destructin-1) that build up as a result of impaired tissue drainage due to reduced blood flow during torpor [60,79,80]. Along with the high concentration of cytotoxic substances, mechanical stress by an increased number of invasive hyphae might also lead to breaches of the cell membrane and local release of pro-death signals [70,72,81]. Whether the fungus nutritionally benefits from this necrotic environment (necrotrophic behavior) or it is counterproductive for its physiological needs remains unknown [69]. During this phase, *P*. *destructans* often breaches the epidermal basement membrane and progresses into the collagen-rich dermis, potentially using the same enzymatic machinery (e.g., Destructin-1) [60,65,79]. This later phase of WNS has been more frequently described than the early invasive phase, with cupping lesions becoming a diagnostic hallmark of WNS and the ultraviolet light fluorescence induced by fungal metabolites (e.g., riboflavin, siderophores) being harnessed for noninvasive diagnosis of WNS and to target *P*. *destructans*-infected skin for in vivo RNA sequencing studies [62,79,80,82–84]. Fungal structures in these cupping lesions are highly pleomorphic with slender and thin-walled superficial hyphae and bulbous, thick-walled hyphae in deeper areas of the lesion like those described under heat stress [38] (**Fig 4**). These morphological changes of *P*. *destructans* in different skin layers might be caused by nutritional or hypoxic stress in highly colonized skin areas or by host-secreted antimicrobial products [85,86]. Alternatively, hyphal cell wall thickening and broadening might be a heat stress response to higher arousal frequency induced by WNS [38,87]. The intense epidermal disruption of the wing membrane during this phase translates into physiological changes, including acidosis (elevated pCO_2_ or reduced bicarbonate levels in blood) from reduced excretion of CO_2_ or increased metabolic rate; hypotonic dehydration, including elevated hematocrit and decreased Na^2+^ concentration secondary to increased evaporative water loss and electrolyte leakage; and hyperkalemia (elevated K^+^ in blood) due to the extracellular shift of potassium secondary to acidosis or the leakage of intracellular K^+^ ions from necrotic cells into the bloodstream [26,88–90].
  One of the most counterintuitive observations in the skin of susceptible Nearctic bats (i.e., *M*. *lucifugus*) with advanced *P*. *destructans* infections is the small number of leukocytes at sites of fungal invasion despite the local up-regulation during arousals of genes encoding innate immune mediators (i.e., alarmins [IL1α, IL1β, TNFα]) involved in activation and recruitment of phagocytes (i.e., neutrophils and macrophages) [62,79,91–93]. Likewise, genes encoding cytokines known to polarize T cells towards a Th17 response (i.e., TGFβ1, IL6, IL23) are up-regulated in the *P*. *destructans*-infected skin without induction of IL-17A (Th17 cytokine product), which is otherwise up-regulated in regional lymph nodes of WNS–positive bats [62,91–93]. These cytokine patterns indicate that local immune and nonimmune cells respond to advanced invasive WNS with proinflammatory mediators and an attempt to shape the host response towards a Th17-type antifungal response. However, proper local innate and adaptive responses might be undermined by the torpor-induced inability of sequestered phagocytes and lymphoid cells to migrate to the skin and the short window of time when these leukocytes are fully functional [14–20].
  Despite the relative paucity of effector cells, locally expressed pyrogenic and proinflammatory cytokines (i.e., IL1α, IL1β, TNFα) are likely systemically released upon arousal, leading to activation of the acute phase response (A2M, C3, PF, and TF), which might lead to an energetically draining febrile state [94–96]. Like during early invasion, COX2 is one of the most up-regulated genes during this later invasive phase [59,62,91,92]. Although untested, proinflammatory COX2 products such as prostaglandins or thromboxanes are likely expressed under these more damaging circumstances [76,77]. In that scenario, PGE_2,_ known to induce arousal in ground squirrels, might be partially responsible for increased arousal frequency during advanced WNS [97]. These immunological changes and the other physiological changes caused by skin barrier disruption (described above) result in increased arousal frequency, which leads to positive feedback loops worsening the physiological imbalance and causing loss of fat stores, starvation, and in many cases, death, which in *M*. *lucifugus* occurs around 88 to 114 days postinfection in captive settings [26,35,88–90,98].
**4) Death or resolution.** Bats with severe WNS that survive until emergence from hibernation present with an overwhelmingly high fungal antigen burden and proinflammatory mediators. Upon reconstitution of the immune response, both innate and adaptive responses become hyperactivated and lead to an immune reconstitution inflammatory syndrome (IRIS), characterized by severe tissue damage and pathology, including exuberant pyogranulomatous inflammatory infiltrates, pustules, abscesses, thrombosis and infarction of skin tissues, or shock [54,99]. Emergent bats that survive the IRIS clear *P*. *destructans*, the necrotic debris, and the degenerated inflammatory infiltrates, replacing them with newly formed epithelium and expelling the debris and degenerated cells onto the skin surface, forming crusts that are finally shed. Deeper lesions involving extensive basement membrane disruption are likely replaced by scar tissue [55].

### *P*. *destructans* interactions with the microbiome

The skin surface hosts microbial communities (microbiome) that establish mostly neutral or beneficial interactions with the host [100]. Diverse microbiomes offer a more protective environment against invasive microbial pathogens [101]. However, microbial diversity in the skin of Nearctic bats is typically determined by the microbial composition of the hibernacula environment and does not correlate with susceptibility to WNS [102]. Conversely, *P*. *destructans*’ colonization of bat skin greatly lowers the skin bacterial (but not fungal) diversity of the highly susceptible little brown bat (*M*. *lucifugus*) but not of other WNS-affected species like the big brown bat (*E*. *fuscus*) and the tricolored bat (*Perimyotis subflavus*) [102]. Despite the lack of correlation between microbiome diversity and susceptibility to WNS, specific components of the microbiome might still inhibit *P*. *destructans* invasion through the production of antimicrobial compounds, modulation of the host immune system, or competition for adherence sites and metabolic niches [103,104]. In this regard, several bacterial and fungal species can inhibit *P*. *destructans*’ growth. For example, bacteria in the genus *Pseudomonas*, commonly found on bat skin, inhibit the growth of *P*. *destructans* both in vitro and in vivo [105–108]. Also, the bacterium *Rhodococcus rhodochrous* and a yeast differentially abundant in WNS-resistant bat species, *Cutaneotrichosporon moniliiforme*, inhibit *P*. *destructans*’ growth in vitro [109,110].

## Factors that influence the severity of WNS

Infection severity is the result of the host’s inherent susceptibility, environmental factors, and the pathogen’s intrinsic virulence, which are factors that collectively modulate host–pathogen interactions [111].

### Host intrinsic factors

#### Immune response

Differences in susceptibility to WNS between bat species and populations have been shown [6]. Palearctic bats like the greater mouse-eared bat (*M*. *myotis*) are not reported to suffer mortalities from WNS, while populations of some Nearctic species, like *M*. *lucifugus*, have plummeted in the last 18 years because of WNS [6]. One of the most remarkable differences between these two related species is *M*. *myotis*’ milder local immune response to *P*. *destructans* invasion [112]. This dampened response could result from developed tolerance (reduced immune reactivity) to *P*. *destructans* [112,113]. However, the small number of *P*. *destructans* invasion sites in *M*. *myotis* indicates that efficient constitutive antimicrobial mechanisms might halt early fungal progression through the skin, precluding extensive epidermal damage and induction of inflammation. Moreover, *M*. *lucifugus* populations that persist after exposure to *P*. *destructans* and a North American bat species less affected by WNS (*E*. *fuscus*) have been found to respond to *P*. *destructans* with gene induction patterns similar to those observed in pre-epidemic naïve *M*. *lucifugus* [112]. It is, therefore, unclear if disparities in the antifungal immune response are critical for survival to WNS, and additional studies are needed to determine whether subtle qualitative and quantitative differences could explain disease outcome.

#### Skin barrier

Besides immunological factors, genomic analysis of WNS-naïve versus persisting *M*. *lucifugus* populations have highlighted a positive selection of genes involved in skin regeneration and wound healing in WNS survivors [114]. As discussed earlier, epidermal damage in the wing membrane likely contributes to physiological disruption and host death. In addition, genes involved in keratinocyte differentiation and wound healing are enriched during the late phase of WNS, likely as an attempt to restore the structural gap caused by the fungus [92]. Therefore, enhanced healing capacity by certain bat species or populations might minimize disruption of skin homeostasis and increase their chances of surviving WNS.

### Ecological factors

#### Inoculum size

Bat colonies can acquire *P*. *destructans* infections by interacting with the contaminated environment. Therefore, it follows that bat populations occupying sites with abundant environmental *P*. *destructans* loads have lower population growth [115]. Overall, the prevalence of *P*. *destructans* in Eurasian hibernacula is lower than in North American caves or mines. In addition, environmental *P*. *destructans* loads in Palearctic hibernacula sharply decrease during the summer, while pathogen loads remain constantly high in Nearctic hibernacula [46]. Consequently, the higher levels of environmental contamination in North American hibernation sites likely translate to higher inoculum size and may explain higher infection rates, earlier disease onset, and higher disease severity and mortality rates by the end of the winter.

#### Relative humidity

As discussed earlier, *P*. *destructans* germination and growth are higher at high RH%, and hibernating bat species that are highly susceptible to WNS select wintering sites with high humidity (typically 60% to 100%) to prevent dehydration [116,117]. Species more affected by WNS are also more susceptible to water loss (e.g., *M*. *lucifugus*, *Myotis septentrionalis*, and *P*. *subflavus*) and select microclimates for hibernation with the highest RH% (often their skin is covered by condensed water), a favorable environment for *P*. *destructans* to thrive [26,116].

#### Clustering and bat density

Communal hibernation and clustering are other behaviors that lead to higher WNS severity. Clustering is frequently observed in highly populated hibernacula, like those of pre-epidemic *M*. *lucifugus* populations. Clustering during hibernation minimizes heat and evaporative water loss but also increases transmission of *P*. *destructans* by causing increased contact between infected and noninfected bats [26,116–118]. Therefore, the lower impact of WNS in roosting sites with lower bat densities in Europe or caves hosting persisting (but decimated) North American populations of *M*. *lucifugus* might be partially explained by a lower transmission rate in sparsely populated hibernacula.

#### Sex-based behavior

Female bats exhibit higher infection rates, higher pathogen burdens, and lower survival rates [119]. In autumn, male bats mate with torpid females, while females prioritize torpor to conserve energy for spring reproduction [13]. Hence, the increased activity of male bats during autumn and their shorter hibernation period likely results in reduced optimal conditions for *P*. *destructans*’ replication and infection.

#### Temperature

Bats that select warmer microclimates closer to the temperature range optimal for *P*. *destructans* growth (12 to 16°C) have higher fungal loads and suffer greater WNS effects than those that select cooler roosts [120]. However, prolonged exposure of *P*. *destructans* to temperatures above 20°C (its upper critical temperature) is unfavorable for the fungus’ growth. Consistent with this, healthy, uninfected big brown bats (*E*. *fuscus*), a species with low WNS-related mortalities, undergo higher arousal frequencies and shorter bouts of torpor during hibernation than those from the susceptible species *M*. *lucifugus*; these longer euthermic periods in *E*. *fuscus* might halt fungal progression and retard pathogenesis [64].

### Pathogen factors

Intrinsic genetic differences in virulence between strains of *P*. *destructans* are not currently considered significant determinants of disease severity. However, this could be due to the small number of strains of *P*. *destructans* that have been used in experimental studies. One experiment in which *M*. *lucifugus* was infected with a *P*. *destructans* isolate from either Europe or North America led to comparable pathology and mortality rates [34,35]. However, many strains of *P*. *destructans* have yet to be assessed in comparative virulence studies. North American isolates of *P*. *destructans* are expanding clonally (i.e., they are essentially genetically homogeneous) and are likely to be similar in virulence [34]. However, future introduction of the MAT1-2 idiomorph might favor sexual reproduction, leading to increased diversity and the emergence of new virulence attributes that favor the adaptation of *P*. *destructans* to the host [121]. The higher plasticity of *P*. *destructans* could lead to less virulent genotypes that are nonpathogenic commensal mutualists or more virulent genotypes that cause even more severe and lethal infections.

## Conclusions and future of WNS pathogenesis research

Since the first report of WNS in 2006, substantial progress has been made in understanding WNS pathogenesis [9,11]. Initial descriptions of WNS cases and isolation of *P*. *destructans* as the presumptive causative agent were followed by experimental infections to confirm Koch’s postulates and to better understand disease progression and host responses in different bat species [8,35,54,62,65,79,91–93,95,112,122]. Because *P*. *destructans* represented the first major animal pathogen within the Leotiomycetes, there was little baseline information on which to infer basic biology, host–pathogen interactions, and fungal virulence mechanisms. A substantial body of research was conducted to address these gaps, including genomic comparisons and in vitro determination of nutritional and environmental needs and enzymatic activity [34,38,41–43,45,61,123]. In vitro characterization of *P*. *destructans*’ secretome and gene expression comparison between nonpathogenic (*P*. *destructans* grown in culture media) and pathogenic (in vivo infection) settings have hinted at potential virulence factors [50,60,62].

These early studies nurtured the field with numerous hypotheses on host–*P*. *destructans* interactions, such as the protective, tolerant response of Palearctic bats versus the immunopathogenic response of Nearctic bats such as *M*. *lucifugus*. Many of these hypotheses remain untested, partly due to limitations to studying these interactions in vivo [112,113]. Some of these limitations are due to (1) technical challenges of reproducing hibernation conditions in captivity that capture the inherent variability found within a natural hibernaculum; (2) limited availability of bats for terminal experiments due to ethical concerns and the legal conservation status of those species; and (3) genetic, epigenetic, and microbiome diversity between populations and individuals that increase variability, statistical error, and reproducibility between experiments.

More recently, in vitro cell culture models of host–pathogen interactions have been developed and validated, providing more reproducible systems that allow fine dissection and understanding of molecular and cellular mechanisms involved in the pathogenesis of WNS [59]. In vitro systems offer many additional advantages in modeling the pathogenesis of WNS. First, in a more controlled in vitro setting, various parameters, such as cell types (e.g., keratinocytes versus leukocytes), fungal life stage and burden, and environmental conditions (e.g., temperature and humidity), can be readily manipulated. Second, in vitro versatility increases experimental resolution and makes it easier to identify dependent variables such as the activation of receptors, expression of fungal ligands, or virulence factors. Third, in vitro models offer a higher reproducibility level while allowing the manipulation of genetic and molecular factors in either the pathogen (e.g., virulence factors) or host cells (key components of host response and susceptibility). Fourth, these models allow rapid testing of many compounds to identify those with promising effects, such as chemical inhibitors of different receptors on pathogen–host interactions. Fifth, they are often more cost-effective given the specialized type of attention needed to work with wild hibernating bats. Lastly, conducting preliminary studies in vitro and only advancing promising and thoroughly validated hypotheses in live bats is ethically more appropriate. This is particularly important given the critical conservation status of most bat species, especially those sensitive to WNS.

Despite the many advantages of in vitro cell culture models, findings from such work would ideally be validated with more complex in vitro and ex vivo models. Although such complex models await development and validation, they might include organoids or stratified skin models with multiple cell types, ex vivo models like skin explants, and immune chimeras (i.e., transfer of bat immune cells to immune-deficient mouse hosts) [124,125]. Finally, in vivo experimental infections in bats, like those described above, or larger field studies could be used to ensure the relevance of the in vitro discoveries at the individual and population levels.

Multiple gaps in the knowledge of WNS pathogenesis warrant further investigation. Some of these are (1) identification of *P*. *destructans* virulence factors; (2) characterization of host immune components and responses involved in pathogenesis; and (3) elucidation of unique features of bat immunometabolism during hibernation.

1. **Identification of *P*. *destructans*’ virulence factors:** Genetic manipulation of *P*. *destructans* using protoplast- or agrobacterium-mediated transformation followed by classic homologous recombination or a high-throughput approach such as CRISPR/Cas9 could be used for identification of virulence factors using a literature-based or unbiased (CRISPR/Cas9 library screening) approach. As discussed earlier, these loss-of-function experiments could be paired with phenotypic screening utilizing in vitro systems and further validated ex vivo and in vivo.
2. **Characterization of host immune components and responses involved in pathogenesis.** Further work identifying receptors and pathways that favor *P*. *destructans* invasion and survival in the host and protective versus deleterious responses to this infection may enable the identification of therapeutic or preventive approaches to manage WNS. Additionally, foundational profiling of skin immune cells and responses from susceptible versus resistant or tolerant bat species or populations using either bulk or single-cell RNA sequencing or proteomics could be instrumental in identifying molecules and pathways that can be rigorously interrogated in in vitro systems followed by in vivo validation.
3. **Elucidation of unique features of bat immunometabolism during hibernation.** Unique features of their biology have shaped bats’ coevolution with microbes. One of these traits is their capacity to hibernate. Open questions on how hibernation influences immune responses to microbes, including fungi, are numerous. Particularly relevant is the likely connection between unique metabolic changes experienced by hibernating bats during the different stages of their life history (torpor versus euthermia) and their antimicrobial responses (immunometabolism).

This proposed endeavor to understand WNS pathogenesis can help lead to groundbreaking discoveries with applications in bat conservation and human and veterinary medicine.
